# RNA Viruses in *Blechomonas* (Trypanosomatidae) and Evolution of *Leishmaniavirus*

**DOI:** 10.1128/mBio.01932-18

**Published:** 2018-10-16

**Authors:** Danyil Grybchuk, Alexei Y. Kostygov, Diego H. Macedo, Jan Votýpka, Julius Lukeš, Vyacheslav Yurchenko

**Affiliations:** a Life Science Research Centre, Faculty of Science, University of Ostrava, Ostrava, Czech Republic; b Department of Parasitology, Faculty of Science, Charles University, Prague, Czech Republic; c Biology Centre, Institute of Parasitology, Czech Academy of Sciences, České Budejovice (Budweis), Czech Republic; d Faculty of Sciences, University of South Bohemia, České Budejovice (Budweis), Czech Republic; e Martsinovsky Institute of Medical Parasitology, Tropical and Vector Borne Diseases, Sechenov University, Moscow, Russia; Duke University; San Diego State University; Max Planck Institute

**Keywords:** *Blechomonas*, *Leishbunyaviridae*, *Leishmaniavirus*, *Narnaviridae*

## Abstract

In this work, we analyzed viral prevalence in trypanosomatid parasites (*Blechomonas* spp.) infecting Siphonaptera and discovered nine species of viruses from three different groups (leishbunyaviruses, narnaviruses, and leishmaniaviruses). Most of the flagellate isolates bore two or three viral types (mixed infections). Although no new viral groups were documented in *Blechomonas* spp., our findings are important for the comprehension of viral evolution. The discovery of bunyaviruses in blechomonads was anticipated, since these viruses have envelopes facilitating their interspecific transmission and have already been found in various trypanosomatids and metatranscriptomes with trypanosomatid signatures. In this work, we also provided evidence that even representatives of the family *Narnaviridae* are capable of host switching and evidently have accomplished switches multiple times in the course of their evolution. The most unexpected finding was the presence of leishmaniaviruses, a group previously solely confined to the human pathogens *Leishmania* spp. From phylogenetic inferences and analyses of the life cycles of *Leishmania* and *Blechomonas*, we concluded that a common ancestor of leishmaniaviruses most likely infected *Leishmania* first and was acquired by *Blechomonas* by horizontal transfer. Our findings demonstrate that evolution of leishmaniaviruses is more complex than previously thought and includes occasional host switching.

## Abstract (precis)

Flagellates belonging to the genus *Leishmania* are important human parasites. Some strains of different *Leishmania* species harbor viruses (leishmaniaviruses), which facilitate metastatic spread of the parasites, thus aggravating the disease. Up until now, these viruses were known to be hosted only by *Leishmania*. Here, we analyzed viral distribution in *Blechomonas*, a related group of flagellates parasitizing fleas, and revealed that they also bear leishmaniaviruses. Our findings shed light on the entangled evolution of these viruses. In addition, we documented that *Blechomonas* can be also infected by leishbunyaviruses and narnaviruses, viral groups known from other insects’ flagellates.

## INTRODUCTION

Trypanosomatidae are a diverse family of flagellates primarily parasitizing insects (1). The vast majority of known trypanosomatids are monoxenous, i.e., restricted to a single (mainly insect) host. However, at least three lineages independently acquired the ability to infect other hosts, such as plants (*Phytomonas* spp.) and vertebrates (*Trypanosoma* spp. and a group that unites *Leishmania*, *Paraleishmania*, and *Endotrypanum*), using insects as vectors (2–5). Because of their medical or economic importance, dixenous species were studied in minute detail, while their monoxenous relatives remained mostly neglected (1, 6). One of such usually disregarded groups—genus *Blechomonas* (subfamily Blechomonadinae)—comprises flagellate parasites of fleas (7). In many phylogenetic reconstructions, this clade is a sister to all other trypanosomatids excluding Trypanosomatinae (*Trypanosoma* spp.) and Paratrypanosomatinae (*Paratrypanosoma* spp.) (8–11). Such a position implies an early origin of this group. Nevertheless, since the genus description in 2013, very little attention has been paid to its members, although the genome of the type species, Blechomonas ayalai, has been sequenced and included in some recent phylogenomic analyses (12, 13).

The importance of dixenous parasites determined their priority in the studies of viruses of trypanosomatids (14, 15). The first-ever characterized virus in these flagellates was *Leishmania RNA virus 1* (LRV1) (16). This is a double-stranded RNA (dsRNA) virus of the family *Totiviridae* found in the New World Leishmania guyanensis (17). LRV1 impedes the immune response against *Leishmania* and facilitates metastatic spread of the parasites (18, 19). A related *Leishmania RNA virus 2* (LRV2) was shown to infect Leishmania major, Leishmania aethiopica, and Leishmania infantum in the Old World (20, 21). It was proposed that *Leishmania* spp. and LRV1/2 have coevolved for a long time (20, 22).

A recent large-scale survey of RNA viruses in trypanosomatids revealed the presence of four other viral groups, confirming some previous unsystematic reports (23–25). These groups are the tombus-like viruses {positive single-stranded RNA [(+)ssRNA] genome, proposed taxon}, leishbunyaviruses (LBVs) [(−)ssRNA genome, proposed taxon], narnaviruses (NVs) [(+)ssRNA or dsRNA genomes, formally recognized family], and an unusual ostravirus (26). Interestingly, no relatives of leishmaniaviruses have been found in the analyzed flagellates, leading to a speculation that LRV1/2 were acquired by an ancestor of modern *Leishmania* and subsequently lost in most extant species. This conclusion was mainly based on the analysis of viral presence in the monoxenous species of the genera *Crithidia* and *Leptomonas*, close phylogenetic relatives of the dixenous *Leishmania*, *Paraleishmania*, and *Endotrypanum* (27). However, many groups of Trypanosomatidae were not included in the screening, rendering this interpretation preliminary.

In this work, we investigated the diversity of viruses in flea-infecting trypanosomatids of the genus *Blechomonas* and report the presence of three different types of viruses in these flagellates, including those related to the prototypical leishmaniaviruses of the family *Totiviridae*.

## RESULTS AND DISCUSSION

### Screening and sequencing.

**FIG 1:**
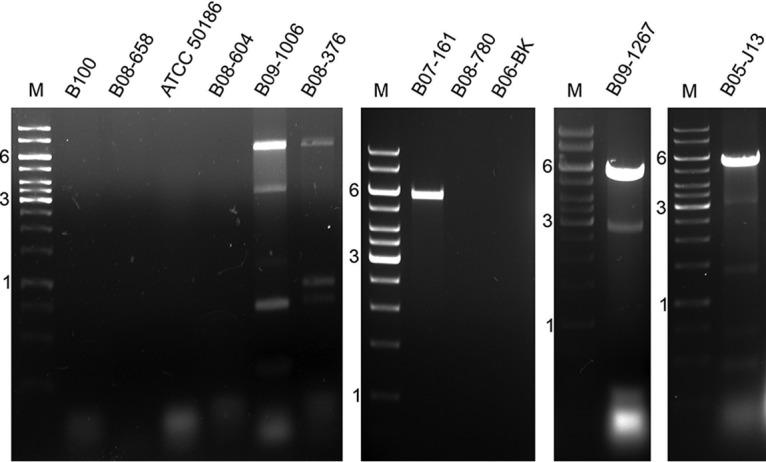
RNA viruses of *Blechomonas* spp.: Blechomonas keelingi B100, B. luni B08-658, B. pulexsimulantis ATCC 50186, B. lauriereadi B08-604, B. luni B09-1006, B. ayalai B08-376, B. juanalfonzi B07-161, B. danrayi B08-780, B. campbelli B06-BK, B. wendygibsoni B09-1267, and B. maslovi B05-J13. M, GeneRuler 1-kb DNA ladder. Indicated sizes are in kilobases. The shortest dsRNA fragment from B. maslovi B05-J13 (∼470 bp) returned no identifiable BLAST hits.

Twelve isolates of *Blechomonas* spp. used in this analysis were described in considerable detail previously (7). The additional strain of Blechomonas luni (B09-1006) available in our collection was isolated from the flea Chaetopsylla globiceps, collected on the red fox Vulpes vulpes in the Czech Republic in 2009 (see Table S1 in the supplemental material). In five isolates (Blechomonas luni B09-1006, B. ayalai B08-376, Blechomonas juanalfonzi B07-161, Blechomonas maslovi B05-J13, and Blechomonas wendygibsoni B09-1267), we documented the presence of the dsRNA bands (Fig. 1). These samples were sequenced using the Illumina HiSeq platform, each yielding 2.4 Gbp of sequence data on average. Sequence analyses revealed that these five isolates contain, in total, nine new viruses from three distinct viral groups (Table 1). Importantly, these groups comprise viruses with (+)ssRNA, (−)ssRNA, and dsRNA genomes, allowing sensitive detection of either their genomes or respective replicative intermediates (in the case of ssRNA). Viral genomic RNA sequences were mostly complete, except for the M segments of B. luni LBV1 (*Blun*LBV1) and B. maslovi LBV1 (*Bmas*LBV1), and the narnavirus *Bmas*NV1, which were incomplete at their 3′ ends. Here, viruses are named according to the established convention indicating an abbreviated host name and viral affiliation (LBV, LRV, or NV for leishbunyaviruses, leishmaniaviruses, and narnaviruses, respectively). Coinfections with more than one virus were documented for three out of five analyzed isolates (Table 1).

**TABLE 1:** Virus-positive *Blechomonas* spp.^*a*^

Host strain	Host and viral RNA	Length (nt)	Accession no.
*B. luni* B09-1006	*Blun*LBV1 L segment	5,974	MG967334
	*Blun*LBV1 M segment	1,059	MG967335
	*Blun*LBV1 S segment	618	MG967336
	*Blun*NV1	2,747	MG967337
*B. ayalai* B08-376	*Baya*LBV1 L segment	6,009	MG967338
	*Baya*LBV1 M segment	822	MG967339
	*Baya*LBV1 S segment	646	MG967340
*B. juanalfonzi* B07-161	*Bjua*LRV4	5,429	MG967341
*B. maslovi* B05-J13	*Bmas*LBV1 L segment	6,251	MG967342
	*Bmas*LBV1 M segment	1,411	MG967343
	*Bmas*LBV1 S segment	706	MG967344
	*Bmas*LRV3	5,412	MG967345
	*Bmas*NV1	2,945	MG967346
*B. wendygibsoni* B09-1267	*Bwen*LRV3	5,403	MG967347
	*Bwen*NV1	2,748	MG967348

a Species, isolate names, and GenBank accession numbers of the identified viral sequences are indicated. LBV, *Leishbunyavirus*; LRV, *Leishmaniavirus*; NV, *Narnavirus*.

10.1128/mBio.01932-18.3 TABLE S1 *Blechomonas* species isolates used in this work. Species, isolate names, GenBank accession numbers for the gGAPDH and 18S rRNA genes, and host specificity are indicated. Download Table S1, DOCX file, 0.01 MB. Copyright © 2018 Grybchuk et al. 2018 Grybchuk et al. This content is distributed under the terms of the Creative Commons Attribution 4.0 International license.

**FIG 2:**
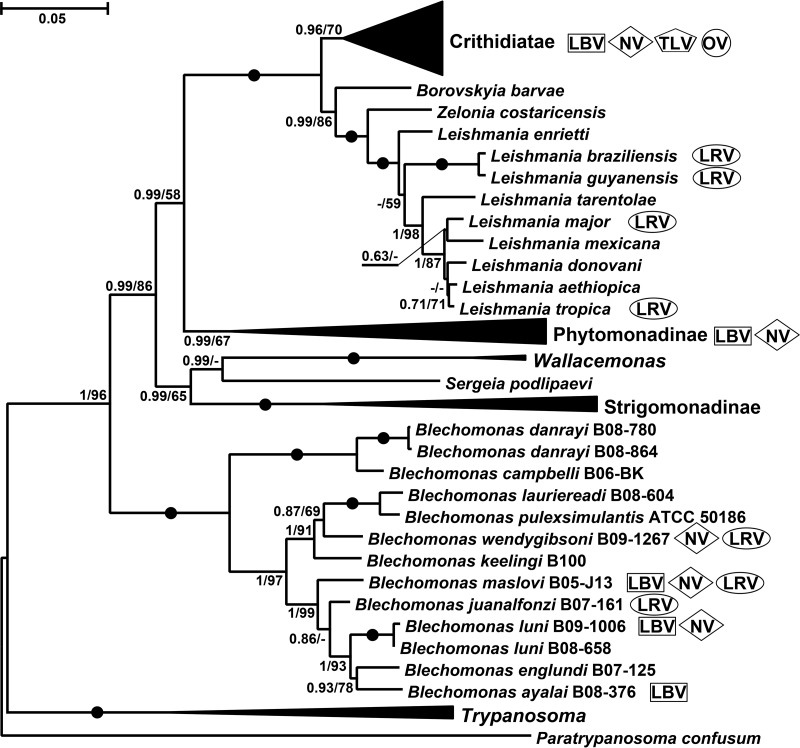
Viruses of Trypanosomatidae. Maximum likelihood phylogenetic tree of trypanosomatids reconstructed using 18S rRNA and gGAPDH genes (78 operational taxonomic units [OTUs]). Viral groups infecting these flagellates are indicated on the right (see key at top). Numbers at nodes indicate bootstrap percentage/posterior probability. Filled circles mark branches with maximal statistical supports. The scale bar indicates the number of substitutions per site.

No association between occurrence of the viruses and the species of flagellates or their primary/secondary hosts was apparent from the data (Table 1; Fig. 2). Moreover, the very closely related isolates B. luni B09-1006 and B08-658 turned out to be virus positive and virus negative, respectively. As noted previously, caution should be exercised when interpreting results of viral presence or absence (26). If the viral load is (very) low, a sample that is in fact a virus-positive sample may appear virus negative.

### LBVs.

**FIG 3:**
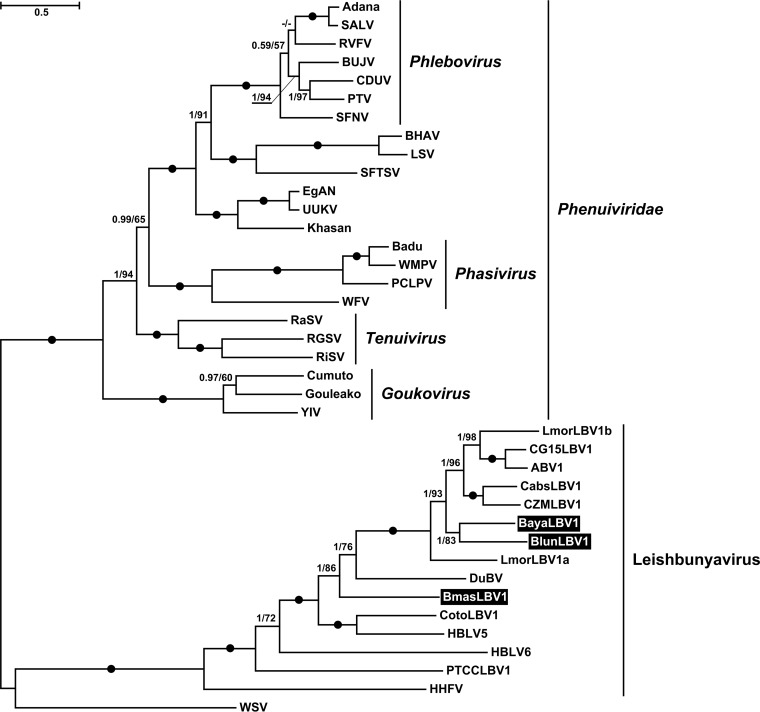
Maximum likelihood phylogenetic tree of proposed *Leishbunyaviridae* based on RDRP amino acid sequences. Numbers at the branches indicate Bayesian posterior probability and maximum likelihood bootstrap supports, respectively; those having a Bayesian posterior probability value of 1.0 and maximum likelihood bootstrap support of 100% are marked with black circles. The scale bar indicates the number of substitutions per site. The tree was rooted with the sequences of *Phenuiviridae*. Abbreviations and GenBank accession numbers are provided in Table S3.

Trypanosomatids are frequently infected with leishbunyaviruses (LBVs) belonging to the recently proposed family *Leishbunyaviridae* of the order *Bunyavirales* (26). In this work, we identified three LBVs infecting different *Blechomonas* spp.—B. luni B09-1006, B. ayalai B08-376, and B. maslovi B05-J13 (Table 1; Fig. 1). All these viruses shared a characteristic tripartite genome arrangement. Their RNA-dependent RNA polymerase (RDRP), nucleocapsid protein, and terminal panhandle sequences were homologous to those of LBVs of Leishmaniinae. The lengths of the M segments, as well as the amino acid sequences of the putative glycoproteins which they encode, were variable. Analysis with transmembrane domain prediction software (TMHMM, TMPred, and Phobius) revealed the presence of at least two transmembrane helices in all putative glycoproteins. Moreover, we have also predicted the N-terminal signal peptide for membrane insertion and N-glycosylation sites using several approaches (SignalP, Signal-BLAST, and Phobius) (Table S2). A similar arrangement of the Leishmaniinae LBV putative glycoproteins had been reported earlier (26). The LBVs of *Blechomonas* (B. ayalai LBV1 [*Baya*LBV1], *Blun*LBV1, and *Bmas*LBV1) were firmly nested within the proposed *Leishbunyaviridae*, although they did not form a single lineage, suggesting at least two independent horizontal transfers, most likely from unrelated trypanosomatids (Fig. 3). At the same time, *Baya*LBV1 and *Blun*LBV1 constitute sister taxa in the obtained tree, and given that their hosts are more closely related to each other than to that of *Bmas*LBV1 (Fig. 2), we believe that it may be an example of virus-flagellate coevolution. The modest number of analyzed isolates does not allow us to generalize this conclusion.

10.1128/mBio.01932-18.4 TABLE S2 Transmembrane domains and signal peptides for *Blechomonas* LBV putative glycoproteins. Download Table S2, DOCX file, 0.01 MB. Copyright © 2018 Grybchuk et al. 2018 Grybchuk et al. This content is distributed under the terms of the Creative Commons Attribution 4.0 International license.

10.1128/mBio.01932-18.5 TABLE S3 Abbreviations and GenBank accession numbers used for phylogenetic inferences. Download Table S3, DOCX file, 0.02 MB. Copyright © 2018 Grybchuk et al. 2018 Grybchuk et al. This content is distributed under the terms of the Creative Commons Attribution 4.0 International license.

### Narnaviruses.

Narnaviruses are capsidless viruses containing a single RDRP-encoding transcript (28–30). Originally, they were found in the yeast Saccharomyces cerevisiae but later were also detected in oomycetes (31) and trypanosomatids (23, 24, 26, 32).

We documented narnaviruses in three trypanosomatid isolates—B. luni B09-1006, B. maslovi B05-J13, and B. wendygibsoni B09-1267 (Fig. 1; Table 1). Interestingly, in the first two cases the corresponding dsRNA bands could not be detected using the DNase I-LiCl method (data not shown), whereas an S1 nuclease-based approach allowed their visualization on the gel (Fig. 1). All three viral RNAs were ∼3.0 kb long and contained a single open reading frame (ORF) encoding RDRP as well as two to three stem-loop structures on both the 5′ and 3′ ends (Fig. S1). In narnaviruses from yeasts, these structures are essential for viral replication and defense against exonucleases of the host (30). It is worth noting that the short terminal complementary sequences in narnaviruses of *Blechomonas* spp. (5′-CCCG…CGGG-3′) differ from the homologous regions in narnaviruses of S. cerevisiae (5′-GGGGGC…GCCCC-3′ [33]).

10.1128/mBio.01932-18.1 FIG S1 Secondary structures and complementary sequences on 5′ and 3′ ends of *Blechomonas* NVs predicted by IPknot. The short terminal complementary sequences are highlighted. Download FIG S1, PDF file, 0.4 MB. Copyright © 2018 Grybchuk et al. 2018 Grybchuk et al. This content is distributed under the terms of the Creative Commons Attribution 4.0 International license.

**FIG 4:**
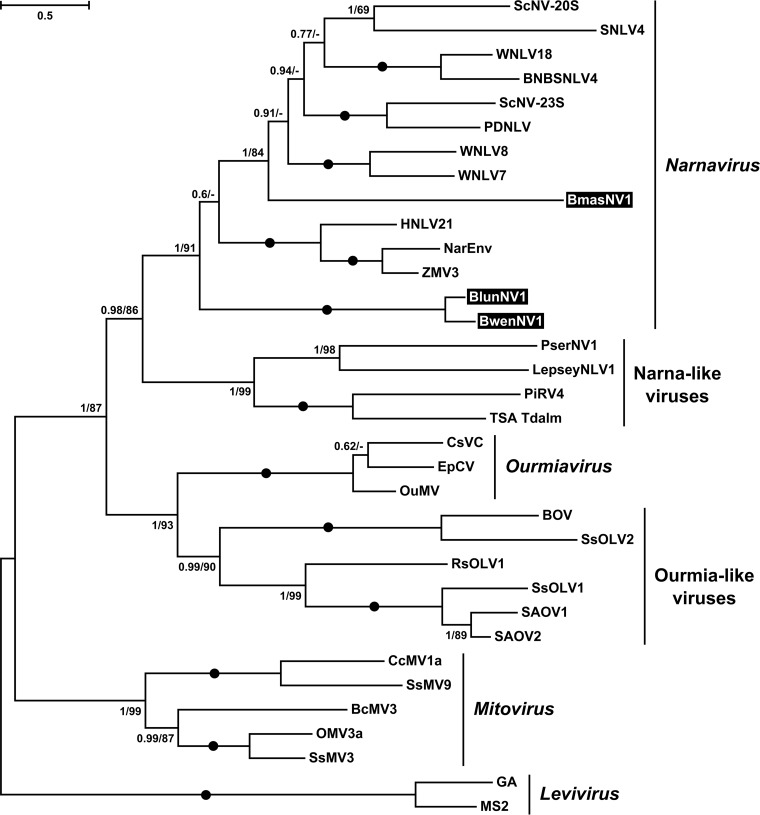
Maximum likelihood phylogenetic tree of *Narnaviridae* based on RDRP amino acid sequences. Numbers at the branches indicate Bayesian posterior probability and maximum likelihood bootstrap supports, respectively; those having a Bayesian posterior probability value of 1.0 and maximum likelihood bootstrap support of 100% are marked with black circles. The scale bar indicates the number of substitutions per site. The tree was rooted with the sequences of *Leviviridae*. Abbreviations and GenBank accession numbers are provided in Table S3.

On the phylogenetic tree (Fig. 4), *Blechomonas* NVs (*Bmas*NV1, B. wendygibsoni NV1 [*Bwen*NV1], and *Blun*NV1) grouped with members of the genus *Narnavirus*, enclosing prototypical 20S and 23S RNA viruses of S. cerevisiae (34) and viruses found in environmental arthropod metatranscriptomes (35). The previously described representatives of *Narnaviridae* infecting trypanosomatids Leptomonas seymouri and Phytomonas serpens were situated in a separate clade of the so-called Narna-like viruses (Fig. 4). This fact along with the nonmonophyletic distribution of narnaviruses from *Blechomonas* spp. suggests that trypanosomatids have acquired these viruses at least three times independently. In addition, a comparison of the phylogenies of trypanosomatids (Fig. 2) and their viruses (Fig. 4) revealed a discrepancy: *Blun*NV1 forms a sister clade to *Bwen*NV1 and *Bmas*NV1 is not closely related to them, whereas B. luni is more closely related to B. maslovi than to B. wendygibsoni. The most parsimonious explanation of this implies a horizontal transfer of viruses between two unrelated flagellate species. Insect hosts are quite often infected by two or more trypanosomatid species (36–38), potentially facilitating such horizontal transfer.

### Leishmaniaviruses: first representatives outside *Leishmania*.

Three out of 13 analyzed isolates (Blechomonas juanalfonzi, B. maslovi, and B. wendygibsoni) were documented to bear viruses of the genus *Leishmaniavirus* (LRV) of the family *Totiviridae*. Their single-RNA genomes contain two overlapping ORFs (+1 ribosomal frameshift) coding for the capsid protein and RDRP (Fig. S2A). The same genomic organization is inherent to leishmanial LRV1s but not LRV2s. The RDRP sequence of the latter virus is either in-frame or in −1 frame relative to the capsid protein (20, 21, 39). A stem-loop structure and a slippery sequence are two structural elements governing the ribosomal frameshift, which were also identified in *Blechomonas* LRVs (Fig. S2A). As in other members of the *Totiviridae*, 3′ termini of *Blechomonas* LRVs were predicted to form stem-loop structures (Fig. S2B). Although not conserved on the sequence level, these *cis* elements had been implicated in replication and RNA packing of the yeast L-A virus (40) and Leishmania guyanensis LRV1 (41).

10.1128/mBio.01932-18.2 FIG S2 *Blechomonas* LRV1 structural features. (A) Frameshifts in capsid and RDRP ORFs. Slippery sequences are in bold, stop codons are in red, and nucleotides which form Watson-Crick and wobble base pairs are underlined. (B) Putative secondary structures at the 3′ termini of *Bjua*LRV1, *Bmas*LRV1, and *Bwen*LRV1. Stem-loop structure-forming bases are underlined; stop codons are in red. A structure of LRV1 to -4 from *Leishmania guyanensis* is shown for comparison. Download FIG S2, PDF file, 0.6 MB. Copyright © 2018 Grybchuk et al. 2018 Grybchuk et al. This content is distributed under the terms of the Creative Commons Attribution 4.0 International license.

**FIG 5:**
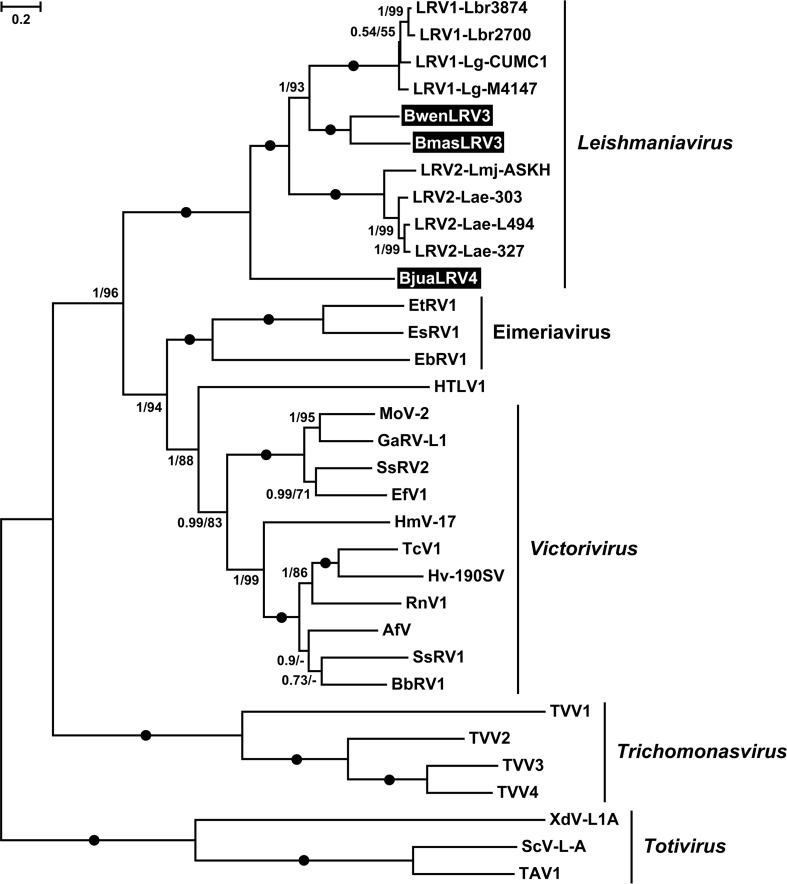
Maximum likelihood phylogenetic tree of *Leishmaniavirus* based on concatenated capsid-RDRP amino acid sequences. Numbers at the branches indicate Bayesian posterior probability and maximum likelihood bootstrap supports, respectively; those having a Bayesian posterior probability value of 1.0 and maximum likelihood bootstrap support of 100% are marked with black circles. The scale bar indicates the number of substitutions per site. The tree was rooted with the sequences of *Trichomonasvirus*. Abbreviations and GenBank accession numbers are provided in Table S3.

**FIG 6:**
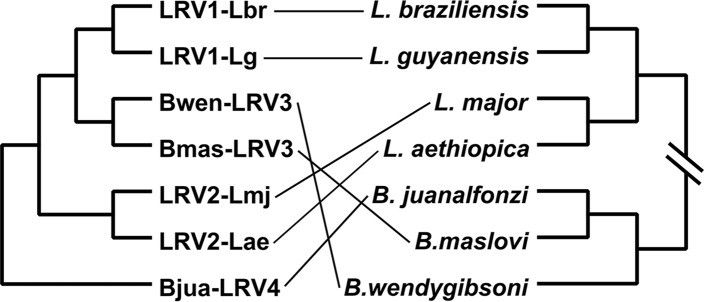
Comparison of the phylogenies of leishmaniaviruses and their respective hosts. The scheme is based on the phylogenetic trees presented in Fig. 2 and 5 with simplifications: (i) no branch lengths are shown on either tree and (ii) only LRVs and LRV-containing trypanosomatids were included.

Phylogenetic analyses using a concatenated capsid-RDRP data set demonstrated a strongly supported monophyly of leishmaniaviruses from *Leishmania* and *Blechomonas* (Fig. 4) and some intermingling of the two groups (Fig. 5), suggesting a horizontal transfer of viruses between the two distantly related trypanosomatid genera. Given that the new viruses are quite distinct from the previously characterized LRV1 and LRV2, we named the leishmaniaviruses from B. wendygibsoni and B. maslovi LRV3s and the virus from B. juanalfonzi LRV4. B. juanalfonzi LRV4 (*Bjua*LRV4) represents the deepest branch in the LRV clade, whereas *Bmas*LRV3 and *Bwen*LRV3 are sisters to the LRV1s from the New World *Leishmania* spp. (Fig. 5 and 6). Similarly to the situation with narnaviruses, there was a significant discrepancy between the phylogenies of the LRVs from *Blechomonas* and their flagellate hosts. *Bwen*LRV3 and *Bmas*LRV3 formed a clade, whereas *Bjua*LRV4 was distant from them. The host of *Bwen*LRV3 was not closely related to those of the two other viruses (Fig. 6). This finding marks the first occurrence of viruses from the genus *Leishmaniavirus* and the family *Totiviridae* in trypanosomatids other than representatives of the genus *Leishmania*.

### Viral coinfections.

**FIG 7:**
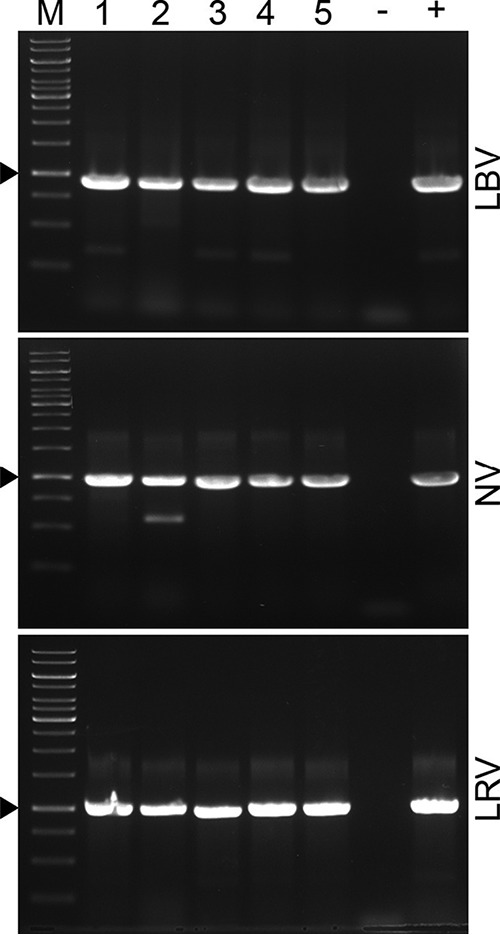
LBV, NV, and LRV presence in clonal lines of Blechomonas maslovi B05-J13. RT-PCR analysis with virus-specific primers. M, GeneRuler 1-kb DNA ladder. Clonal lines are numbered from 1 to 5. Triangles denote specific PCR products. −, negative PCR control; +, primary culture of B. maslovi B05-J13 used as a positive control.

To understand whether coinfecting viruses infect all or just subsets of cells in a given population, we analyzed viral infection in isolated clonal cell lines. As a model, B. maslovi B05-J13 was used to generate clones, because its primary culture was simultaneously infected with three different viruses, namely, LBV, LRV, and NV (Table 1). Our results demonstrate that all obtained clones invariably harbored all three viruses (Fig. 7), confirming this triple infection on the level of single cells.

### Conclusions.

The recent survey of viral diversity in trypanosomatids, which are composed of 52 isolates belonging to ∼20 species from three genera, documented 13 species of RNA viruses and demonstrated that besides the well-studied LRVs, at least four other groups of viruses occur in these flagellates (26). Here, with a relatively modest sampling (13 isolates of 11 species, belonging to a single genus) we were able to discover a comparable number of viruses: nine species from three different groups (leishbunyaviruses, narnaviruses, and leishmaniaviruses). The high number of the new discovered viruses is explained by mixed viral infections in some isolates of *Blechomonas* spp., representing novel “hotbeds” of viral discovery, as was Leptomonas pyrrhocoris in our previous study. Whether the presence of viruses in *Blechomonas* is harmful or beneficial to their hosts, e.g., in the interplay with their insect vectors, remains to be investigated further.

Although no new viral groups were documented in *Blechomonas* spp., our findings are important for the comprehension of viral evolution. The discovery of LBVs in blechomonads was anticipated, since these viruses have envelopes facilitating their interspecific transmission and have already been found in various trypanosomatids and metatranscriptomes with trypanosomatid signatures (26). As in the previous study, in the case of *Baya*LBV1 and *Blun*LBV1, we documented potential lateral transfer of viruses and short-term virus-trypanosomatid coevolution.

The new findings concerning narnaviruses demonstrated that their ability for host switching was significantly underestimated (42). Previously, it was considered that owing to the simple organization of these viruses (single RNA coding only for RDRP), they could be transmitted only vertically or during mating (29). Therefore, we have proposed that Leptomonas seymouri and Phytomonas serpens inherited narnaviruses from a common ancestor, while many other trypanosomatids lost them (26). However, all narnaviruses found in *Blechomonas* spp. were unrelated to those documented in other trypanosomatids and did not form a monophyletic clade by themselves. In addition, a horizontal transfer of viruses is a parsimonious explanation for the sister relationships of the distantly related *Blun*NV1 and *Bwen*NV1. Thus, we provided evidence that even “naked” viruses are capable of host switching and evidently have accomplished switches multiple times in the course of their evolution. The endocytosis via flagellar pocket of trypanosomatids (43–45) is a plausible route of the acquisition of narnaviruses.

Although none of the three viral groups documented in the *Blechomonas* hosts is new, the presence of LRVs was unexpected, since until now, they were confined solely to the human pathogens *Leishmania* spp. (26). The discovery of LRVs in monoxenous trypanosomatids unrelated to *Leishmania* sheds new light on the origin and evolution of these viruses. As suggested by the phylogenetic analyses, members of the genus *Leishmaniavirus* apparently originated from the fungal viruses, which represent a regular component of the intestinal microbiome of insects (46, 47). It is tempting to propose an early divergence of *Bjua*LRV4 as evidence of blechomonads being the first hosts of LRVs, yet deducing a common ancestor of this genus from the phylogenetic tree remains problematic. Indeed, a simple parsimony analysis shows that regardless of whether a given *Leishmania* or *Blechomonas* species is selected as an ancestral host, the number of intergeneric transitions remains the same, namely, two. In order to reconstruct the evolution of leishmaniaviruses and, in particular, pinpoint their possible transitions between the *Leishmania* and *Blechomonas* hosts, it is important to consider the life cycles of these flagellates and their insect hosts and propose plausible scenarios, in which parasites could meet and exchange their viruses. For *Leishmania* spp., this is well studied: during blood-feeding of a sandfly on an infected vertebrate host, the parasites enter the gut, where they propagate and then migrate to the anterior part and are transmitted to another vertebrate during the next blood meal (48, 49). However, the life cycles of monoxenous trypanosomatids are largely unknown, but in general, the described routes of transmission include feeding on a contaminated substrate, direct coprophagy, necrophagy, and vertical transmission through eggs’ surfaces (1, 45, 50, 51).

While the life cycles of *Blechomonas* spp. were never studied, it has been proposed that the infection of fleas occurs at the larval stage and persists into the imago stage (7). Indeed, adult fleas are strictly hematophagous and therefore cannot acquire any pathogen by regular means (52). The flea larvae are scavengers consuming dead insect bodies, conspecific eggs, detritus from host nests, feces of adult fleas, etc., and thus may acquire flagellates by coprophagy and necrophagy. In addition, there should be a transphasic transmission of the protists into adults, i.e., their preservation during metamorphosis (7). Taking into account all these factors, one can only speculate about where *Leishmania* and *Blechomonas* meet. This cannot be the sandfly’s gut, since there is no way for a *Blechomonas* to enter it. It is also unlikely to occur in the blood of a vertebrate, since blechomonads along with their viruses would be quickly eliminated by the immune system before sharing their viruses with *Leishmania* residing in the highly specific compartment of phagolysosomes of macrophages. In our opinion, the flea gut is the most likely place for such an exchange. Indeed, trypanosomatids can survive in a nonspecific insect host for a considerable period (32, 53), and even *Leishmania* parasites were detected in adult fleas (54). Both *Leishmania* and *Blechomonas* may have enough time for contacts, and they are separated by no barriers in both imagos and larvae. Under these circumstances, leishmanias are doomed and can serve only as donors of viruses. The adult fleas may obtain *Leishmania* from the blood of an infected vertebrate, while their larvae may become infected after consuming feces of the adults, which are full of partially digested blood (55, 56), or from dead bodies of infected adult sandfly females dying at their breeding grounds, e.g., rodent nests, which are common for both sandfly and flea larvae. According to the presented scenarios, only transmissions of viruses from *Leishmania* to *Blechomonas*, but not vice versa, may occur. It was previously proposed that *Leishmania* spp. coevolved with LRVs for a long time (22). Our findings demonstrate that the evolution of LRVs is much more complex and includes host switching. A recent discovery of an LRV2 in Leishmania infantum (57) suggests that horizontal transfers might occur also between different *Leishmania* species.

## MATERIALS AND METHODS

### Parasite culture, DNA and RNA isolation, and molecular marker analysis.

The cultures of Blechomonas ayalai, B. campbelli, B. danrayi, B. englundi, B. juanalfonzi, B. keelingi, B. lauriereadi, B. luni, B. maslovi, B. pulexsimulantis, and B. wendygibsoni (a total of 13 isolates [see Table S1 in the supplemental material]) were initially grown on biphasic blood agar overlaid with RPMI 1640 medium (Thermo Fisher Scientific, Waltham, USA) for 1 to 3 weeks. For DNA and RNA isolation, *Blechomonas* spp. were subpassaged in brain heart infusion (BHI) medium (Sigma-Aldrich, St. Louis, MO, USA) supplemented with 10 µg/ml of hemin (Jena Bioscience GmbH, Jena, Germany), 10% fetal bovine serum (FBS), 500 units/ml of penicillin, and 0.5 mg/ml of streptomycin (all from Thermo Fisher Scientific) as reported previously (9). DNA was isolated from 5 × 10^7^ cells using the Qiagen DNeasy Blood & Tissue kit (Qiagen, Hilden, Germany) and amplified with primers M200 and M201 (for glycosomal glyceraldehyde-3-phosphate dehydrogenase [gGAPDH]) or S762 and S763 (for 18S rRNA), as described previously (58, 59). PCR products were gel purified and sequenced at Macrogen Europe (Amsterdam, The Netherlands). Total RNA was isolated from 0.4 × 10^9^ to 1 × 10^9^ cells as described previously (26).

### dsRNA isolation and next-generation sequencing.

The dsRNA fraction was isolated from 200 µg of total RNA using the DNase-S1 nuclease or DNase I-LiCl method (26) and visualized on an 0.8% agarose gel. RiboMinus libraries were sequenced using Illumina HiSeq 2500 (Illumina, San Diego, CA, USA) at Macrogen Inc. (Seoul, South Korea).

### Viral genome assembly.

Reads were quality checked with FastQC v0.11.5 (60), trimmed with Trimmomatic v0.36 (61), and assembled *de novo* with Trinity v2.4.0 (62). Reads were mapped back to the contigs using Bowtie 2 v2.2.9 (63), sorted with SAMtools v1.3 (64), and viewed in Artemis genome browser v1.8 (65). The “per-base” coverage was calculated using BEDTools program v2.25 (66). Contigs containing viral RNA-dependent RNA polymerase (RDRP) and leishbunyavirus nucleocapsid protein genes were recovered by TBLASTN searches (67). The M segments of leishbunyaviruses were found by visual inspection of read coverage of obtained contigs. The borders of viral sequences within the contigs were delineated by the presence of conserved sequence elements (complementary terminal sequences and/or secondary structures), and in the case of their absence, a cutoff of 10 reads per base was applied.

### Computational analyses. (i) Trypanosomatids.

The trypanosomatid phylogeny was reconstructed using a concatenated 18S rRNA plus gGAPDH gene data set. The core alignments of both genes were taken from a previous study (68), and the groups of interest (*Leishmania* and *Blechomonas*) were expanded. The 18S rRNA gene alignment was purged of poorly aligned positions with Gblocks 0.91b  as described previously (69). Maximum likelihood analysis of the concatenated alignment was performed in IQ-TREE v. 1.5.5 (70) with a partitioning scheme considering genes and codon positions in the gGAPDH gene. The built-in ModelFinder (71) selected the following partitioned model: TPM2u + I + G4, GTR + I + G4, and K3Pu + I + G4 for the first, second, and third codon positions of the gGAPDH gene, respectively, and TNe + I + G4 for the 18S rRNA gene. The branch support was assessed with the use of standard bootstrap method (1,000 replicates). Bayesian inference was accomplished in MrBayes 3.2.6 (72) as described elsewhere (73) with a slight modification of the partition model: GTR + I + G, GTR + G, and GTR + I + G for the three respective codon positions of the gGAPDH gene and GTR + I + G for the 18S rRNA gene.

### (ii) Viruses.

Phylogenetic reconstructions were carried out using the RDRP protein alignments for *Bunyavirales* and *Narnaviridae* and concatenated capsid-plus-RDRP protein alignments for *Totiviridae*. Amino acid sequences were aligned with MAFFT (v. 7.243) using the “E-ins-I” iterative refinement method (74) and trimmed with TrimAl (v. 1.3) with “automated1” settings (75). The scheme of phylogeny reconstruction and the software used were the same as in the case of trypanosomatids (see above). The best-fit models selected by ModelFinder were rtREV + F + I + G4 for *Narnaviridae,* LG + F + I + G4 for *Bunyavirales*, and LG + F + G4 and LG + F + I + G4 for capsid and RDRP of *Totiviridae*, respectively. Bayesian inferences for *Narnaviridae* and *Bunyavirales* were performed using mixed amino acid model prior, which resulted in the 1.0 posterior probability for the Blosum model. Heterogeneity over sites in both cases was estimated under the I + G model. For the *Totiviridae* data set, a partitioned model (LG + I + G, LG + I + G) with unlinked parameters and branch lengths was used. Abbreviations and GenBank accession numbers for viruses used in phylogenetic inferences are listed in Table S3.

Predictions of the transmembrane domains, membrane-targeting signal peptides, and N-glycosylation sites were made in TMHMM (v. 2.0) (76), TMPred (77), Phobius (78), SignalP (v. 4.1) (79), and Signal-BLAST (80).

### Cloning of *B. maslovi*, cDNA synthesis, and screening for viruses by reverse transcription-PCR (RT-PCR).

A primary culture of B. maslovi B05-J13 (simultaneously coinfected by three different viruses) was cloned by limiting dilution as described previously (10). Total RNA (from 4 × 10^8^ cells) was purified using TRIzol (Thermo Fisher Scientific) method (81). RNA was treated with DNase I prior to cDNA synthesis using the Super Script IV first-strand synthesis system (Thermo Fisher Scientific) with random hexamers according to the manufacturer’s protocol.

Viral presence was detected by RT-PCR with the following specific primers: for *Bmas*LBV1, *Bmas*LBV-F 5′-CTAGACTGAGCCCTGATTTC-3′ and *Bmas*LBV-R 5′-ATAACTCGGAATGGTTCTCG-3′ (expected product size 897 bp); for *Bmas*NV1, *Bmas*NV-F 5′-AGTGATCCATTCCGATGATC-3′ and *Bmas*NV-R 5′-AGTCCAAAGTACGAAAGGTC-3′ (expected product size 961 bp); for *Bmas*LRV3, *Bmas*LRV3-F 5′-GCAATTAAGTTCCGACATGG-3′ and *Bmas*LRV3-R 5′-CCAGTTTTTGACTTGGTGTC-3′ (expected product size 980 bp).
