# Supplementary material for: RNA Viruses in Blechomonas (Trypanosomatidae) and Evolution of Leishmaniavirus
Source: mBio. 2018 Oct 16;9(5):e01932-18. doi: 10.1128/mBio.01932-18 (PMC6191543; doi:10.1128/mBio.01932-18)
Supplement: TABLE S1 [file mbo005184111st1.docx]

| **Species** | **Isolate** | **18S** | **gGAPDH** | **Primary host** | **Secondary host** | **Reference** |
| --- | --- | --- | --- | --- | --- | --- |
| *B. ayalai* | B08-376 | KF054116 | KF054093 | *Ctenophthalmus agyrtes* | Rodentia (nest) | Votypka et al. 2013 |
| *B. keelingi* | B100 | KF054131 | KF054109 | *Ceratophyllus pullatus* | *Parus major* | Votypka et al. 2013 |
| *B. luni* | B08-658 | MH055749 | KF054103 | *Chaetopsylla globiceps* | *Vulpes vulpes* | Votypka et al. 2013 and this work |
| *B. luni* | B09-1006 | MH055750 | MH064438 | *Chaetopsylla globiceps* | *Vulpes vulpes* | this work |
| *B. pulexsimulantis* | ATCC 50186 | KF054128 | KF054090 | *Pulex simulans* | *Canis lupus* f. *domesticus* | Votypka et al. 2013 |
| *B. campbelli* | B06-BK | KF054133 | KF054097 | *Ctenocephalides felis* | *Felis silvestris* f. *catus* | Votypka et al. 2013 |
| *B. danrayi* | B08-780 | KF054135 | KF054095 | *Chaetopsylla globiceps* | *Vulpes vulpes* | Votypka et al. 2013 |
| *B. danrayi* | B08-864 | KF054136 | MH064439 | *Chaetopsylla globiceps* | *Vulpes vulpes* | Votypka et al. 2013 and this work |
| *B. englundi* | B07-125 | KF054118 | KF054101 | *Monopsyllus sciurorum* | *Sciurus vulgaris* | Votypka et al. 2013 |
| *B. juanalfonzi* | B07-161 | KF054121 | KF054099 | *Ctenophthalmus* sp. | *Microtus arvalis* | Votypka et al. 2013 |
| *B. lauriereadi* | B08-604 | KF054127 | KF054100 | *Ctenocephalides canis* | *Vulpes vulpes* | Votypka et al. 2013 |
| *B. maslovi* | B05-J13 | KF054123 | KF054104 | *Paraceras melis* | *Meles meles* | Votypka et al. 2013 |
| *B. wendygibsoni* | B09-1267 | KF054126 | KF054092 | *Nycteridopsylla eusarca* | *Nyctalus noctula* | Votypka et al. 2013 |
