# Supplementary material for: RNA Viruses in Blechomonas (Trypanosomatidae) and Evolution of Leishmaniavirus
Source: mBio. 2018 Oct 16;9(5):e01932-18. doi: 10.1128/mBio.01932-18 (PMC6191543; doi:10.1128/mBio.01932-18)
Supplement: TABLE S3 [file mbo005184111st3.docx]

| **Clade** | **Short name** | **Virus name** | **RDRP Accession** | **Coat Accession** |
| --- | --- | --- | --- | --- |
| **LRV-like viruses** | | | | |
| *Victorivirus* | AfV | Aspergillus foetidus slow virus 1 | [CCD33024.1](https://www.ncbi.nlm.nih.gov/protein/400131542?report=genbank&log$=prottop&blast_rank=13&RID=PCEEAZBR014) | [CCD33023.1](https://www.ncbi.nlm.nih.gov/protein/400131541?report=genbank&log$=prottop&blast_rank=47&RID=PCEE0E89015) |
| *Victorivirus* | BbRV1 | Beauveria bassiana victorivirus 1 | [AMQ11131.1](https://www.ncbi.nlm.nih.gov/protein/1005516319?report=genbank&log$=prottop&blast_rank=23&RID=PCEEAZBR014) | [AMQ11130.1](https://www.ncbi.nlm.nih.gov/protein/1005516318?report=genbank&log$=prottop&blast_rank=48&RID=PCEE0E89015) |
| Eimeriavirus | EbRV1 | Eimeria brunetti RNA virus 1 | [AAK26438](https://www.ncbi.nlm.nih.gov/protein/AAK26438) | [AAK26437](https://www.ncbi.nlm.nih.gov/protein/AAK26437) |
| Eimeriavirus | EsRV1 | Eimeria stiedai RNA virus 1 | [AOM63180.1](https://www.ncbi.nlm.nih.gov/protein/1062509343?report=genbank&log$=prottop&blast_rank=62&RID=PCEEAZBR014) | [AOM63179.1](https://www.ncbi.nlm.nih.gov/protein/1062509342?report=genbank&log$=prottop&blast_rank=44&RID=PCEE0E89015) |
| Eimeriavirus | EtRV1 | Eimeria tenella RNA virus 1 | [AIW58883](https://www.ncbi.nlm.nih.gov/protein/AIW58883) | [AIW58882](https://www.ncbi.nlm.nih.gov/protein/AIW58882) |
|  | HTLV1 | Hubei toti-like virus 1 | APG76077.1 | APG76077.1 |
| *Leishmaniavirus* | LRV1-Lbr2700 | Leishmania braziliensis LEM2700 Leishmania RNA virus 1 | APU54690 | [APU54689](https://www.ncbi.nlm.nih.gov/protein/APU54689) |
| *Leishmaniavirus* | LRV1-Lbr3874 | Leishmania braziliensis LEM3874 Leishmania RNA virus 1 | APU54696 | [APU54695](https://www.ncbi.nlm.nih.gov/protein/APU54695) |
| *Leishmaniavirus* | LRV1-Lg-CUMC1 | Leishmania guyanensis CUMC1 Leishmania RNA virus 1 | [AAB50024](https://www.ncbi.nlm.nih.gov/protein/331420) | [AAB50023](https://www.ncbi.nlm.nih.gov/protein/AAB50023) |
| *Leishmaniavirus* | LRV1-Lg-M4147 | Leishmania guyanensis M4147 Leishmania RNA virus 1 | [AAB50028](https://www.ncbi.nlm.nih.gov/protein/515646) | [AAB50027](https://www.ncbi.nlm.nih.gov/protein/AAB50027) |
| *Leishmaniavirus* | LRV2-Lae-303 | Leishmania aethiopica LDS303 Leishmania RNA virus 2 | AHK06414 | AHK06413 |
| *Leishmaniavirus* | LRV2-Lae-327 | Leishmania aethiopica LDS327 Leishmania RNA virus 2 | AHK06416 | AHK06415 |
| *Leishmaniavirus* | LRV2-Lae-L494 | Leishmania aethiopica LRC-L494 Leishmania RNA virus 2 | AHZ10902 | AHZ10901 |
| *Leishmaniavirus* | LRV2-Lmj-ASKH | Leishmania major 5-ASKH Leishmania RNA virus 2 | [AAB50031](https://www.ncbi.nlm.nih.gov/protein/1045467) | [AAB50030](https://www.ncbi.nlm.nih.gov/protein/AAB50030) |
| *Victorivirus* | EfV1 | Epichloe festucae virus 1 | [CAK02788.1](https://www.ncbi.nlm.nih.gov/protein/94536500) | [CAK02787.1](https://www.ncbi.nlm.nih.gov/protein/94536499) |
| *Victorivirus* | GaRV-L1 | Gremmeniella abietina RNA virus L1 | [AAK11656.1](https://www.ncbi.nlm.nih.gov/protein/13022153) | [AAK11655.1](https://www.ncbi.nlm.nih.gov/protein/13022152) |
| *Victorivirus* | HmV-17 | Helicobasidium mompa totivirus 1-17 | [BAC81754.1](https://www.ncbi.nlm.nih.gov/protein/33636351) | [BAC81753](https://www.ncbi.nlm.nih.gov/protein/BAC81753) |
| *Victorivirus* | Hv-190SV | Helminthosporium victoriae virus 190S | [AAB94791](https://www.ncbi.nlm.nih.gov/protein/AAB94791) | [AAB94790](https://www.ncbi.nlm.nih.gov/protein/AAB94790) |
| *Victorivirus* | MoV-2 | Magnaporthe oryzae virus 2 | [BAF98178](https://www.ncbi.nlm.nih.gov/protein/BAF98178) | [BAF98177](https://www.ncbi.nlm.nih.gov/protein/BAF98177) |
| *Victorivirus* | RnV1 | Rosellinia necatrix victorivirus 1 | [BAM36400.1](https://www.ncbi.nlm.nih.gov/protein/401664038?report=genbank&log$=prottop&blast_rank=32&RID=PCEEAZBR014) | [BAM36399.1](https://www.ncbi.nlm.nih.gov/protein/401664036?report=genbank&log$=prottop&blast_rank=67&RID=PCEE0E89015) |
| *Victorivirus* | SsRV1 | Sphaeropsis sapinea RNA virus 1 | [AAD11601](https://www.ncbi.nlm.nih.gov/protein/AAD11601) | [AAD11600.1](https://www.ncbi.nlm.nih.gov/protein/3808224) |
| *Victorivirus* | SsRV2 | Sphaeropsis sapinea RNA virus 2 | [AAD11603](https://www.ncbi.nlm.nih.gov/protein/AAD11603) | [AAD11602](https://www.ncbi.nlm.nih.gov/protein/AAD11602) |
| *Victorivirus* | TcV1 | Tolypocladium cylindrosporum virus 1 | [CBY84992](https://www.ncbi.nlm.nih.gov/protein/CBY84992) | [CBY84991](https://www.ncbi.nlm.nih.gov/protein/CBY84991) |
| *Trichomonasvirus* | TVV1 | Trichomonas vaginalis virus 1 | [AAA62868.1](https://www.ncbi.nlm.nih.gov/protein/699096?report=genbank&log$=prottop&blast_rank=71&RID=PCEEAZBR014) | [AAA62867.1](https://www.ncbi.nlm.nih.gov/protein/699095) |
| *Trichomonasvirus* | TVV2 | Trichomonas vaginalis virus 2 | [AED99810.1](https://www.ncbi.nlm.nih.gov/protein/332015894?report=genbank&log$=prottop&blast_rank=85&RID=PCEEAZBR014) | [AED99809.1](https://www.ncbi.nlm.nih.gov/protein/332015893) |
| *Trichomonasvirus* | TVV3 | Trichomonas vaginalis virus 3 | [AKE98372.1](https://www.ncbi.nlm.nih.gov/protein/816270995?report=genbank&log$=prottop&blast_rank=93&RID=PCEEAZBR014) | [AKE98371.1](https://www.ncbi.nlm.nih.gov/protein/816270994) |
| *Trichomonasvirus* | TVV4 | Trichomonas vaginalis virus 4 | [AED99794.1](https://www.ncbi.nlm.nih.gov/protein/332015870?report=genbank&log$=prottop&blast_rank=92&RID=PCEEAZBR014) | [AED99793.1](https://www.ncbi.nlm.nih.gov/protein/332015869) |
| *Totivirus* | ScV-L-A | Saccharomyces cerevisiae virus L-A | AAA50321.1 | AAA50320.1 |
| *Totivirus* | TAV1 | Tuber aestivum virus 1 | ADQ54106.1 | ADQ54105.1 |
| *Totivirus* | XdV-L1A | Xanthophyllomyces dendrorhous virus L1A | AFH09412.1 | AFH09411.1 |
| **Leishbunyaviruses** | | | | |
| *Phlebovirus* | CDUV | Chandiru virus | AEA30057.1 |  |
| *Phlebovirus* | PTV | Punta Toro phlebovirus | ALL45372.1 |  |
| *Phlebovirus* | BUJV | Bujaru virus | API68880.1 |  |
| *Phlebovirus* | Adana | Adana virus | AJK91618.1 |  |
| *Phlebovirus* | SALV | Salehabad phlebovirus | AGA82741.1 |  |
| *Phlebovirus* | SFNV | Sandfly fever Naples virus | CAA48478.1 |  |
| *Phlebovirus* | RVFV | Rift Valley fever virus | ABD51507.1 |  |
|  | SFTSV | Severe fever with thrombocytopenia virus | ADZ04470.1 |  |
|  | BHAV | Bhanja virus | AFO66272.1 |  |
|  | LSV | Lone Star virus | AGL50921.1 |  |
| *Phlebovirus* (uukuniemi group) | EgAN | EgAN 1825-61 virus | AEL29654.1 |  |
| *Phlebovirus* (uukuniemi group) | UUKV | Uukuniemi phlebovirus | BAA01590.1 |  |
| *Phlebovirus* (uukuniemi group) | Khasan | Khasan virus | AII79370.1 |  |
| *Phasivirus* | Badu | Badu phasivirus | AMA19446.1 |  |
| *Phasivirus* | WMPV | Wutai mosquito phasivirus | AJG39270.1 |  |
| *Phasivirus* | PCLPV | Phasi Charoen-like phasivirus | AKP18602.1 |  |
| *Phasivirus* | WFV | Wuhan fly phasivirus | AJG39259.1 |  |
| *Tenuivirus* | RaSV | Ramu stunt virus | ALJ83282.1 |  |
| *Tenuivirus* | RGSV | Rice grassy stunt tenuivirus | BAA89602.1 |  |
| *Tenuivirus* | RiSV | Rice stripe tenuivirus | AFM93792.1 |  |
| *Goukovirus* | Cumuto | Cumuto virus | AHH60917.1 |  |
| *Goukovirus* | Gouleako | Gouleako virus | AEJ38175.1 |  |
| *Goukovirus* | YIV | Yichang Insect virus | AJG39273.1 |  |
| Leishbunyaviridae | ABV1 | Apis bunyavirus 1 | ARO50045.1 |  |
| Leishbunyaviridae | CG15LBV1 | Crithidia sp. G15 leishbunyavirus 1 | ASN64747.1 |  |
| Leishbunyaviridae | LmorLBV1b | Leptomonas moramango leishbunyavirus 1b | ANJ59513.1 |  |
| Leishbunyaviridae | CZMLBV1 | Crithidia sp. ZM leishbunyavirus 1 | ASN64749.1 |  |
| Leishbunyaviridae | CabsLBV1 | Crithidia abscondita leishbunyavirus 1 | AOA33725.1 |  |
| Leishbunyaviridae | LmorLBV1a | Leptomonas moramango leishbunyavirus 1a | ANJ59510.1 |  |
| Leishbunyaviridae | DuBV | Duke bunyavirus | ARE30258.1 |  |
| Leishbunyaviridae | HBLV5 | Hubei bunya-like virus 5 | APG79301.1 |  |
| Leishbunyaviridae | CotoLBV1 | Crithidia otongatchiensis leishbunyavirus 1 | ASN64748.1 |  |
| Leishbunyaviridae | HBLV6 | Hubei bunya-like virus 6 | APG79326.1 |  |
| Leishbunyaviridae | PTCCLBV1 | Phytomonas sp. TCC231 leishbunyavirus 1 | AUF41956.1 |  |
| Leishbunyaviridae | HHFV | Huangshi Humpbacked Fly virus | AJG39239.1 |  |
| Leishbunyaviridae | WSV | Wuhan Spider virus | AJG39269.1 |  |
| **Narnaviruses** | | | | |
| Ourmia-like viruses | SAOV1 | Soybean-associated ourmiavirus 1 | ALM62238 |  |
| Ourmia-like viruses | SAOV2 | Soybean-associated ourmiavirus 2 | ALM62250 |  |
| Ourmia-like viruses | SsOLV1 | Sclerotinia sclerotiorum ourmia-like virus 1 | ALD89138 |  |
| Ourmia-like viruses | RsOLV1 | Rhizoctonia solani ourmia-like virus 1 | ALD89131 |  |
| Ourmia-like viruses | BOV | Botrytis ourmiavirus | CEZ26310 |  |
| Ourmia-like viruses | SsOLV2 | Sclerotinia sclerotiorum ourmia-like virus 2 | ALD89139 |  |
| *Ourmiavirus* | CsVC | Cassava virus C | ACI03053 |  |
| *Ourmiavirus* | EpCV | Epirus cherry virus | ACF16357 |  |
| *Ourmiavirus* | OuMV | Ourmia melon virus | ACF16360 |  |
| *Narnavirus* | ScNV-20S | Saccharomyces cerevisiae 20S RNA narnavirus | AAC98925 |  |
| *Narnavirus* | ScNV-23S | Saccharomyces cerevisiae 23S RNA narnavirus | AAC98708 |  |
| *Narnavirus* | NarEnv | Narnaviridae environmental sample | AJT39596 |  |
| *Narnavirus* | SNLV4 | Shahe narna-like virus 4 | APG77180 |  |
| *Narnavirus* | WNLV8 | Wenling narna-like virus 8 | APG77263 |  |
| *Narnavirus* | WNLV7 | Wenling narna-like virus 7 | APG77259 |  |
| *Narnavirus* | HNLV21 | Hubei narna-like virus 21 | APG77148 |  |
| *Narnavirus* | WNLV18 | Wuhan insect virus 18 | APG77101 |  |
| *Narnavirus* | ZMV3 | Zhejiang mosquito virus 3 | ASA47450 |  |
| *Narnavirus* | PDNLV | Point-Douro narna-like virus | ASA47306 |  |
| *Narnavirus* | BNBSNLV4 | Barns Ness breadcrumb sponge narna-like virus 4 | ASM94069 |  |
| Narna-like viruses | PserNV1 | Phytomoas serpens narnavirus 1 | AUF41955.1 |  |
| Narna-like viruses | LepseyNLV1 | Leptomonas seymouri narna-like virus 1 | ASN64762.1 |  |
| Narna-like viruses | PiRV4 | Phytophthora infestans RNA virus 4 | AEM89291 |  |
| Narna-like viruses | TSA: Tdalm | Teleopsis dalmanni transcribed RNA | GBBP01132666.1 | |
| *Mitovirus* | CcMV1a | Cryphonectria cubensis mitovirus 1a | AAR01970 |  |
| *Mitovirus* | SsMV9 | Sclerotinia sclerotiorum mitovirus 9 | AHF48625 |  |
| *Mitovirus* | BcMV3 | Botrytis cinerea mitovirus 3 | CEZ26302 |  |
| *Mitovirus* | OMV3a | Ophiostoma mitovirus 3a | CAA06228 |  |
| *Mitovirus* | SsMV3 | Sclerotinia sclerotiorum mitovirus 3 | AGC24232 |  |
| *Levivirus* | GA | Enterobacteria phage GA | CAA27499 |  |
| *Levivirus* | MS2 | Enterobacteria phage MS2 | P00585 |  |
