# Supplementary material for: RNA Viruses in Blechomonas (Trypanosomatidae) and Evolution of Leishmaniavirus
Source: mBio. 2018 Oct 16;9(5):e01932-18. doi: 10.1128/mBio.01932-18 (PMC6191543; doi:10.1128/mBio.01932-18)

B. luni B09-1006 NV 5’

ccccgggggggggagcgguuggguucaguacccggccaaacccgcgcgagugaucgcgcggggggcccaccuucugccgaaaaguuucagcagacuugcaggccuaugccguggucuugguacccg
accuggucuagaaaggcugaucc

```
1      ccccgggggggggagcgguuggguucaguacccggccaaacccgcgcgagugaucgcgcggggggcccaccuucugccgaaaaguuucagcagacuugca
1      .....((((((((...((((((((.....))))))))...(((((((((....)))))))))...))).))))).(((...((((((....))))))...
101    ggcc
101    ))).
```

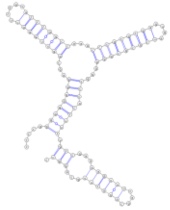

B. luni B09-1006 NV 3’

uuccgguuuuucgagagaccuucucccaaauggaauuuggagacccguggaguacggguaguucggucacagugucuucguuucuguggcccgagcaggagacuaacuggcguuagccuagga
ggggggcuccccccccuccgccuaccuacggg

```
1      aguacggguaguucggucacagugucuucguuucuguggcccgagcaggagacuaacuggcguuagccuaggaggggggcuccccccccuccgccuaccu
1      .....(((((((((((((((((...........))))))).))))).((...(((((.[[.)))))]]..(((((((((....))))))))).)))))))
101    acggg
101    .....
```

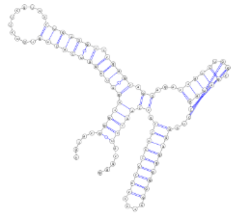

B. wendygibsoni B09-1267 NV 5’

ccgggaggggggagcggucgggcuaacaccccggcuguagguccauggagaaacuccauggaccgagcccacuguguuccgaaaagaauucgcgaacuugcaggccuaugccguggucuuaguau
ccggccua

```
1      ccgggaggggggagcggucgggcuaacaccccggcuguagguccauggagaaacuccauggaccgagcccacuguguuccgaaaagaauucgcgaacuug
1      ...((....(((..((((((((.......))))))))..((((((((((...))))))))))...))).(((((((((((((.....)))).)))))..)
101    caggccu
101    ))).)).
```

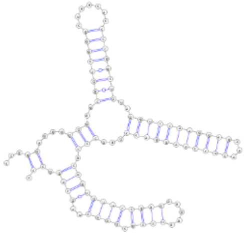

B. wendygibsoni B09-1267 NV 3’

cuggaguagagguaauccaggucgcagugucuucguuucugcaacccugggaggggacugacaggcgucagcccaggcaugggcccccccaugccgccuacccucggg

```
1      cuggaguagagguaauccaggucgcagugucuucguuucugcaacccugggaggggacugacaggcgucagcccaggcaugggcccccccaugccgccua
1      ................((((((.((((...........)))).)).))))...(((.((((((....)))))))).((((((((....)))))))).....
101    cccucggg
101    ........
```

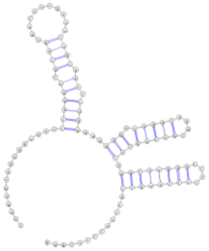

B. maslovi B05-J13 NV 5’

cccgaagggggagagggucaaauuuggucguggagagcgcugucccgacuacucgcccgauucguuucuguucgggcgccuuguuuucuucuuuuaucau

1 cccgaagggggagagggucaaauuuggucguggagagcgcugucccgacuacucgcccgauucguuucuguucgggcgccuuguuuucuucuuuuaucau
1 ....((.[[[....(((((.(((........))))))))...(((((((...........)))))))))]]].................

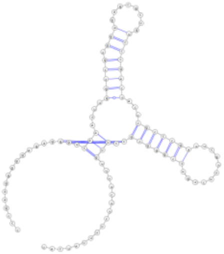

B. maslovi B05-J13 NV 3’

cgggcgaaaauucgagagggcauccgcgguaucaaugaagaaaaaguuuucaaggagucgguuccgacccucguugaugaggcuuuacuagaggguuuuggccucuccaccggcgaauacggu
gaaacguuacguuucuaccgggcuuguccggguuagucg

1 caaggagucgguuccgacccucguugaugaggcuuuacuagaggguuuuggccucuccaccggcgaauacggugaaacguuacguuucuaccgggcuugu
1 ...((((.[[((..]]((((((.......[[.........))))))....))]])))).((((((....((((((((((...)))))).))))....)).
101 ccgggu**uag**ucg
101))))........

Note: The stop codon of RDRP ORF is downstream of predicted complementary terminal sequence

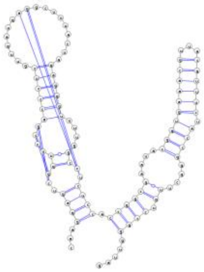

Supplement: FIG S1 [file mbo005184111sf1.pdf]
