# Supplementary material for: RNA Viruses in Blechomonas (Trypanosomatidae) and Evolution of Leishmaniavirus
Source: mBio. 2018 Oct 16;9(5):e01932-18. doi: 10.1128/mBio.01932-18 (PMC6191543; doi:10.1128/mBio.01932-18)

A

Blechomonas juanalfonzi B07-161 LRV1 frameshift

Capsid   L S G T E T V V I E G G S –
cugaguggaacugaaacugugguca uu**gaaggg***g*gcucu**uaa**gacuauccuagccggggaugagga
RDRP – V E L K L W S L K G A L K T I L A G D E

Model of compact H-type pseudoknot

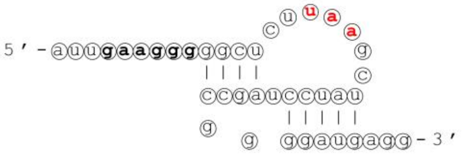

Model of +1 frameshift

```
  E   G                          E   G
 CUU|CCC|                      |CUU|CCC|
 ≡== ≡≡≡           +1            == ≡≡≡
U|GAA|GGG|GG       →          UG|AAG|GGG|G
```

Pseudoknot predicted by Ipknot software

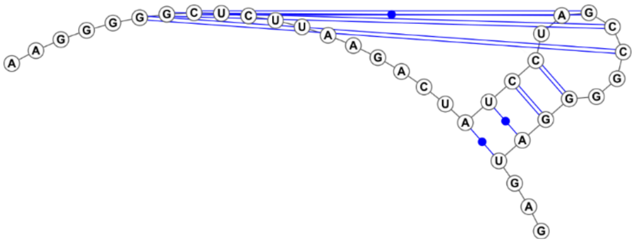

Pseudoknot predicted by Kinefold software

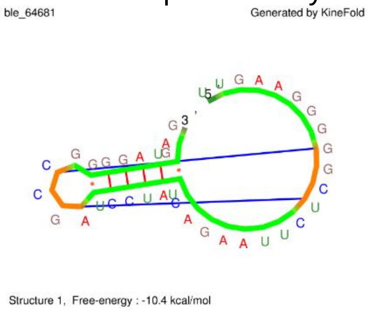

B. maslovi B05-J13 LRV1 frameshift

capsid
S D D N T T E T –
uaagug**aug**acaacacgaca**gaaacu uaa**aacucuguuagacauugu
– V **M** T T R Q K L K T L L D I

B. wendygibsoni B09-1267 LRV1 frameshift

capsid
G R G A V M E P D T E V P A P S A D –
ggucgcggugcaguaauggaaccugacacugagguuccagcaccaagugcugau**uga**caugccuauuuuuaaaggcuguccc
– W N L T L R F Q H Q V L I D M P I L K A V

B

Blechomonas juanalfonzi B07-161 LRV1 3‘ terminus

CAUCACCGUUGCGGCCACUAUUCAUAGUGGCCGCACCCGCUAGGACUUCAGCAACUCCUGGCAGACCGGUCCACCUA**UAA**CCUGGUAUA
CAGCAAUAUAGGUUUUAUGCGUAGCUGACGCAUACAGUAGGCCCG

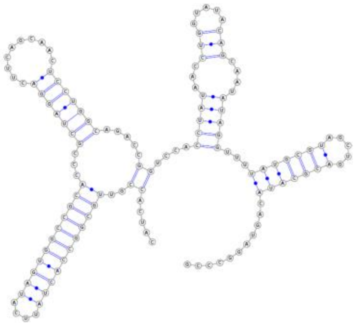

B. maslovi B05-J13 LRV1 3‘ terminus

AUCAUUAAAGUCCUAAACAAUAUUUACGUU**UAG**GCAUAGGCACAAAGUGCCGGAGUGCGAAAGGCG

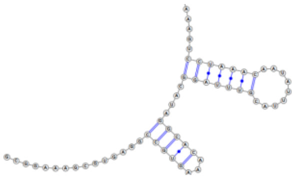

B. wendygibsoni B09-1267 LRV1 3‘ terminus

CUGAGAUUAAAUAUUGUGCUAUUGAUGGUGCACUACCUUAUAGUGAUGCGUGUAUGUUAGCGCGUCGUUUACCAUGUAAUG
GUGCGCUUAUCACUGUAAGACAUAAUAUAUAUACA**UAA**CAAAUUAGACACCGAAUGGUGUCGAUGUGAUUAU

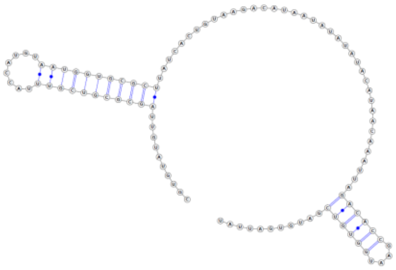

Leishmaia LRV1-4 3’ terminus

GUGGAGUUGUUAUACAAGUAAUACAUAAUGUAUAUGUA**UAA**GGACGCACCAUUCGGAAUAUGGCAAGAGUGCCAUAAACUAUC

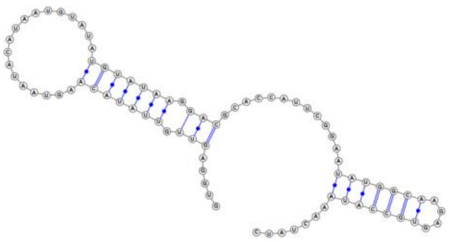

Supplement: FIG S2 [file mbo005184111sf2.pdf]
